# New Energy Utilization Rate and Coal Energy Economic Development Based on the Fuzzy Network Algorithm

**DOI:** 10.1155/2022/1752090

**Published:** 2022-08-27

**Authors:** Cong Wang, Yufei Xu

**Affiliations:** ^1^School of Economics, Ocean University of China, Qingdao 266000, China; ^2^College of Mechanical and Electrical Engineering, China University of Petroleum, Qingdao 266000, China

## Abstract

Energy is an important foundation for the normal development and operation of society. With the rapid development of human society, energy consumption is increasing, energy supply is becoming more and more tenser, and energy consumption is excessively dependent on traditional fossil energy, which will cause serious pollution to the environment. In this regard, this article combines the actual needs of wireless sensor network nodes with limited energy consumption and conducts extensive research on the utilization rate of new energy in a city. By viewing related literature, relevant information on the Internet are collected and collated and then comprehensively analyzed. The main research purpose of this paper is to explore the law of new energy utilization rate in a certain city, determine the methods and paths that can realize the large-scale utilization of new energy, and provide reference materials for urban construction managers. Next, based on the study of coal energy, this article learns that the coal energy output of a certain province accounts for more than 99% of the total energy output. During the period of industrial restructuring, long-term industrial development was carried out under the traditional large-scale model. The development model of high energy consumption and high pollution had a serious impact on the ecological environment, leading to economic development at the expense of the environment in exchange for economic growth. The model is no longer applicable to the existing economic system. This article uses wireless sensing-based research on the utilization of new energy and applies it to the study of coal energy economic development, aiming to promote its better development.

## 1. Introduction

The development and utilization of energy have played an important role in promoting the development of human society. From the appearance of steam engines to the application of intelligent large-scale power systems, they have promoted the development of human society [1]. The rapid development of science and technology in the world today is mainly dependent on energy, especially the exploitation of traditional fossil energy such as oil and coal [2]. However, the storage of traditional fossil energy is limited. With the continuous development and progress of human society, the demand for traditional fossil energy also continuous increase and has gradually caused various problems in human society. All the root causes are caused by the traditional fossil energy supply [3]. As the demand for energy increases, there will be energy shortages or military conflicts caused by competition for energy, which may become more violent [4]. At the same time, the exploitation of traditional fossil energy has caused ecological damage to a certain extent, resulting in serious environmental pollution and threatening human development and survival [5]. This article first analyzes new energy research trends at home and abroad, determines research ideas and methods, and then briefly analyzes the development status of the energy industry, and then introduces the current status and goals of China's energy development, leads to the current status and goals of energy development in a certain city, and uses wireless transmission [6]. Sense technology strategically analyzes the current situation of urban new energy utilization, formulates various energy development policies, constructs urban energy development scenario models, combines scenario models with air pollutant emission reduction, and then analyzes, and finally puts forward corresponding suggestions and countermeasures for the development of new energy utilization in a city [7]. Next, this article analyzes coal energy, the basic national conditions of coal-based petrochemical energy reserves, which determine China's coal-based energy consumption structure and energy production structure and will not change in a long time. However, long-term large-scale mining and use of coal not only caused serious environmental pollution but also caused a lot of waste of coal resources [8]. Taking the road of circular economy development is one of the important ways to solve the comprehensive utilization of coal resources and create an ecological civilization [9]. Based on the system evaluation method, this paper adopts the 3*E* model to analyze the coal industry system of a certain province through the study of circular economy theory, sustainable development theory, energy economic environment theory, and other related theories, constructing the coal industry system coordination evaluation system, and formulating relevant indicators, processing initial data, estimating index coefficients, compiling evaluation procedures, etc., and then conducting a holistic study of the coal industry system in a certain province and discovering its existing problems [10].

## 2. Materials and Methods

### 2.1. Theoretical Basis

The cluster head-based network divides all nodes into different clusters through a hierarchical idea. The nodes in the cluster send information to the cluster heads in one direction, and the cluster heads exchange information with each other. This hierarchical structure loads the energy of the system because it is concentrated in the cluster head, when a new cluster head is periodically elected according to the remaining energy of the node, the energy load of the entire system can be averaged to each node, thereby extending the life of the system.

Before the arrival of the new time, the cluster head must elect a new cluster head and transmit the posterior information of the target state at the current time [11]. The new cluster head integrates the measurement information of all nodes in the cluster to recalculate the posterior of the target state. This structure allows the cluster head's travel route to be synchronized with the target's trajectory [12]. During the filtering and tracking process, the cluster head gradually accumulates the measurement information of all nodes in the cluster. At the same time, the correct selection of the nodes in the cluster consumes relatively few resources at each moment. So the application prospect is broad [13].

**Figure 1:**
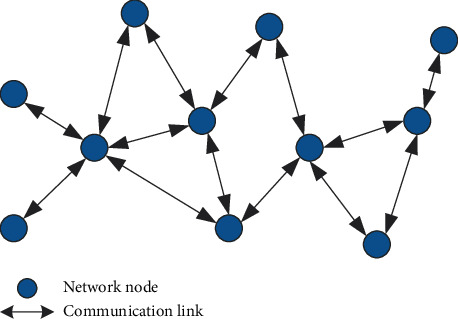
Schematic diagram of the consensus-based network structure.

The consensus-based network structure requires all sensor nodes in the environment to remain active until the tracking task is completed. Each node in the active state can be considered as a fusion center, which continuously receives measurement information from neighboring nodes. After the nodes receive the information, the consensus algorithm decides the next action [14]. After a certain period of time, all nodes obtain the global estimation result of the target state. Specifically, at a certain moment, each node receives the measurement information of neighboring nodes, performs initial state estimation, and independently calculates the local trajectory of the target, and then the local node and neighboring nodes exchange statistical parameters of this trajectory with each other and iteratively execute the specific consensus algorithm until the estimation results of all nodes are unified [15]. At this point, the current time ends and the system enters the next moment.  Figure 1 shows the structure of the consensus network.

In Figure 1, the solid circle represents the activated detection node, and the two-way solid arrow represents the two-way communication link. Obviously, the consensus-based network structure only exchanges information between neighboring nodes without preconfigured routing paths. Local nodes only need to know the characteristics of neighboring nodes, the global estimation results are stored in each node, which makes them particularly suitable for sensor networks composed of mobile nodes that have been widely studied in recent years. However, because the neighboring nodes of the system need to repeatedly send information each time, with the expansion of the network scale, the communication traffic of the entire system network has exploded, and due to the influence of the specific consensus algorithm, the detection cycle of the system is not stable enough. Factors can cause real-time problems.

The consensus algorithm plays an important role in the consensus-based sensor network structure. In the following description, *u*(.) is used to represent the system state update function. Based on three typical consensus algorithms, average consensus, random chat, and maximum consensus, the state function has three different formulas.

Average consensus (BC consensus):

(1) $$\begin{array}{c} \varsigma_{k}^{\left(i\right)}=u\left(\varsigma_{k}^{\left(i-1\right)},{\left\{\varsigma_{k^{\prime }}^{\left(i-1\right)}\right\}}_{k^{\prime }\in {\mathbb{N}}_{k}}\right)=\omega_{k,k}^{\left(i-1\right)}\varsigma_{k}^{\left(i-1\right)}+\sum_{k\in {\mathbb{N}}_{k}}\omega_{k,k^{\prime }}^{\left(i-1\right)}\varsigma_{k^{\prime }}^{\left(i-1\right)}. \end{array}$$

Random chat:

(2) $$\begin{array}{c} \varsigma_{k}^{\left(i\right)}=u\left(\varsigma_{k}^{\left(i-1\right)},{\left(\varsigma_{k^{\prime }}^{\left(i-1\right)}\right)}_{k^{\prime }\in {\mathbb{N}}_{k}}\right)=\frac{1}{2}\varsigma_{k}^{\left(i-1\right)}+\frac{1}{2}\varsigma_{k^{\prime }}^{\left(i-1\right)}. \end{array}$$

The biggest consensus:

(3) $$\begin{array}{c} \varsigma_{k}^{\left(i\right)}=u\left(\varsigma_{k}^{\left(i-1\right)},{\left\{\varsigma_{k^{\prime }}^{\left(i-1\right)}\right\}}_{k^{\prime }\in {\mathbb{N}}_{k}}\right)=\max\left\{\varsigma_{k}^{\left(i-1\right)},\left\{\varsigma_{k^{\prime }}^{\left(i-1\right)}k^{\prime }\in {\mathbb{N}}_{k}\right\}\right\}. \end{array}$$

### 2.2. Research Methods

Traditional fossil energy produces carbon during the combustion process. Carbon emissions from energy consumption activities are an important source of China's carbon emissions and account for the vast majority of China's carbon emissions.

Refer to How to Estimate Carbon Emissions in the United Nations Intergovernmental Panel on Climate Change, and set out how to estimate carbon emissions in this article. In this article, carbon emissions refer to the carbon emissions released by different energy consumption and the carbon emission coefficients of different energy sources in a period of time. The sum of the carbon emissions generated by consuming different energy sources during the period is calculated. The calculation formula is as follows as shown in the following equation:

(4) $$\begin{array}{c} C_{j}^{i}=\sum E_{j}^{i}\times n_{j}. \end{array}$$

In order to simplify and unify the charging method, we have introduced the concept of pollutant equivalent in the design of the charging standard. The pollutant equivalent is specified based on the environmental hazards, biological toxicity, and technical economy of various pollutants or pollutant discharge activities. A quantitative relationship of pollutants or pollutant discharge activities, the formula for calculating the equivalent number is as follows:

(5) $$\begin{array}{c} D_{i}=W_{i}÷d_{i}. \end{array}$$

### 2.3. Application of the Fuzzy Network Algorithm in Energy Economy Calculation

Fuzzy neural network is structured. At present, researchers divide it into five layers, namely, input layer, fuzzy layer, regular layer, synthesis layer, and output layer. Fuzzy subset nodes are contained in the membership functions of the second and fourth layers, and the number of nodes is the number of fuzzy rules. In other words, the rule layer is the library of fuzzy rules.

The first layer is the input layer. The main function of this layer is to convert each input node into two functions, namely, synthesis function *F* (·) and activation function *A* (·), in which synthesis function *F* (·) can sort out the information of connected nodes and activation function *A* (·) can sort out the corresponding activation value. In this formula, we use *NN*_1_ to represent the number of neurons, which will be numerically equal to the number of input variables or nodes:

(6) $$\begin{array}{c} f_{k}=u_{k}^{\left(1\right)},\\ a_{k}=f_{k}\;\left(1\leq k\leq NN_{1}\right), \end{array}$$

where the connection weights of this layer are set to be equal to 1, and *U*_*K*_^(1)^ is equal to the size of the kth input variable.

The second layer is the fuzzy layer. The number of neurons is represented as *NN*_2_, which depends on *NN*_1_ and its corresponding fuzzy subset number.

(7) $$\begin{array}{c} f_{k}=\frac{{\left(u_{i}^{\left(2\right)}-m_{ij}^{\left(2\right)}\right)}^{2}}{{\left(\sigma_{ij}^{\left(2\right)}\right)}^{2}},\;a_{k}=e^{f_{k}}\left(1\leq k\leq NN_{2}\right), \end{array}$$

where the relationship between *i*, *j*, and *k* is as follows:

(8) $$\begin{array}{c} i=\frac{\left(k-1\right)}{N_{2}}+1,\\ j=\left(k-1\right)\%N_{2}+1. \end{array}$$

The third layer is the rule layer. Fuzzy rule number is also called the node number, so the rule layer we often say is fuzzy rule library. The conditions and conclusions of the rule nodes are derived from the network connections between layer 2 and layer 3 nodes and the network connections between layer 3 and layer 4 nodes, respectively.

(9) $$\begin{array}{c} f_{k}=\underset{1\leq j\leq NN_{1}}{\min}\left(u_{kj}^{\left(3\right)}\right),\\ a_{k}=f_{k}\;\left(1\leq k\leq NN_{3}\right), \end{array}$$

where the connection weights of this layer are set to be equal to 1.

The fourth layer is the comprehensive layer. The number of neurons in this layer, *NN*_4_, must meet a prerequisite, and *NN*_4_ = *NN*_5_ × *N*_5_. *NN*_5_ is the number of output variables, where the number of fuzzy subsets corresponding to each variable is represented by *N*_5_ in this paper.

(10) $$\begin{array}{c} f_{k}=\sum_{j=1}^{N_{4k}}u_{kj}^{04},a_{k}=\min\left(1,f_{k}\right)\;\left(1\leq k\leq NN_{4}\right). \end{array}$$

Make the connection weights of this layer equal to 1.

The fifth layer is the output layer. This layer mainly obtains a clear value through membership function.

(11) $$\begin{array}{c} f_{k}=\sum_{j=1}^{N_{5}}\left(m_{kj}^{\left(5\right)}*\sigma_{kj}^{\left(5\right)}\right)*u_{kj}^{\left(5\right)},\\ a_{k}=\frac{f_{k}}{\sum_{j=1}^{N_{s}}\sigma_{kj}^{\left(5\right)}\times u_{kj}^{\left(5\right)}}\;\left(1\leq k\leq NN_{5}\right). \end{array}$$

In these two formulas, we need to pay attention to the fuzzy subset membership function, especially its center and width are easily confused, so that the output of a clear value is wrong, so this paper uses *M*_*KJ*_^(5)^ and *σ*_*kJ*_^(5)^, respectively.

When we look at the TFP analysis, we divide it into many frames, and there are many elements in these frames. Total factor energy efficiency index is measured by the target energy input required to output the same amount of economic output/actual energy input under optimal production conditions. Energy efficiency is shown in formula (13):

(12) $$\begin{array}{c} EE_{i,t}=\frac{AEI_{i,t}-LEI_{i,t}}{AEI_{i,t}}=\frac{TEI_{i,t}}{AEI_{i,t}}. \end{array}$$

Based on the fuzzy neural network, this paper conducts measurement analysis on energy economic efficiency and establishes the following SBM model to measure energy economic efficiency:

(13) $$\begin{array}{c} \min EEC=\frac{1-\left(1/m\right)\sum_{i=1}^{m}s_{i}^{-}/x_{i0}}{1+\left(1/s\right)\sum_{r=1}^{s}s_{r}^{+}/y_{r0}}\begin{array}{c} x_{0}=X\lambda +s^{-},y_{0}=Y\lambda -s^{+},\\ \lambda \geq 0,s^{-}\geq 0,s^{+}\geq 0, \end{array} \end{array}$$

where the relaxation value of the *i*th and *r* input index is represented by *S*_*i*_^+^ and *S*_*r*_^+^, respectively.

## 3. Results

### 3.1. New Energy Development and the Utilization Level

**Figure 2:**
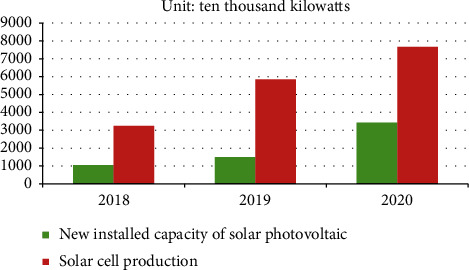
2018–2020 New PV installed capacity and PV cell output.

Investors' investment confidence and producers' production decisions are determined to a certain extent by the quality of the market environment, and consumers' consumption choices are also determined by the quality of the market environment. A good market environment requires not only government management but also all market participants to regulate their own behavior and benefit from it fairly and legally. At present, China is obviously not sufficiently aware of the strategic, long-term, and complex nature of renewable energy and new energy market construction. Also lack of awareness of the high development costs of nonrenewable and new energy and renewable energy and new energy still lack widespread social recognition and a sound market environment in China. In addition, insufficient power grid construction capabilities will eventually lead to China's new energy the industry and its market scale cannot effectively cope with it, and the development speed of the new energy market is much lower than that of the new energy industry. As shown in Figure 2, China's newly installed solar photovoltaic capacity and solar photovoltaic cell output have shown an increasing trend from 2018 to 2020, but the new installed solar photovoltaic capacity has always been lower than the industrial capacity of solar photovoltaic cell production, and its own digestion capacity is not strong.

At the same time, the current energy industry still protects the traditional fossil energy industry and restricts the development of new energy industries. The fair development environment of the new energy industry is not optimistic.

**Figure 3:**
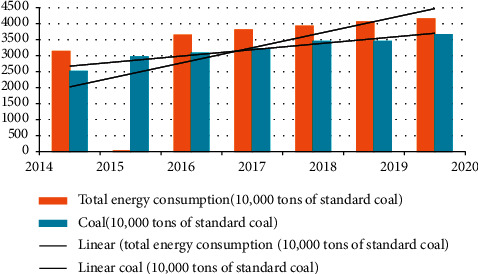
Energy consumption of a city from 2010 to 2020.

As shown in Figure 3, the annual growth rate of energy consumption and total energy consumption in a city from 2014 to 2020 is relatively stable, and the energy consumption structure is dominated by coal consumption. Since 2014, coal consumption has accounted for 80% of total energy consumption and it will reach 88% in 2020.

### 3.2. Energy Industry Output Value Processing

**Figure 4:**
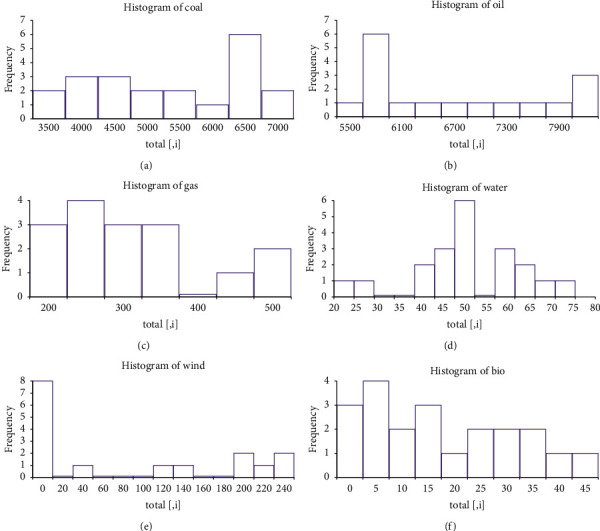
Histogram of the frequency distribution of various energy sources from 2001 to 2020. (a) Histogram of coal. (b) Histogram of oil. (c) Histogram of gas. (d) Histogram of water. (e) Histogram of wind. (f) Histogram of bio.

As shown in Figure 4, the distribution of data is judged based on the histogram of the output value of each energy industry.

Table 1 shows the total contribution rate of each new energy source to GDP from 2001 to 2020.

It can be seen from the regression results that the energy industry occupies a relatively high position in terms of the national economic aggregate. Compared with the traditional energy industry, the new energy industry has a better traction rate, and its contribution to economic growth is greater and more stable. Among the several types of new energy industries mentioned, we can know the GDP contribution of the past few years. In most years, the contribution rate of the wind energy industry is relatively low, indicating that wind energy is not suitable for large-scale promotion in a certain province. Economic development is not very useful. Over the years, the hydropower industry has shown a large economic contribution, but the facts proved that the contribution is not stable. It may be due to the annual climate and environmental differences. The contribution rate of biomass energy has shown a relatively stable state each year. As an agricultural industry in a large province, there is a surplus of stable biomass in a certain province every year. Although the utilization of biomass energy is stable, it can be said that it is not fully utilized. This stable contribution shows that the biomass energy industry still has great development prospects. However, as mentioned in the previous comparative analysis, compared with the other two new energy industries, biomass energy is definitely suitable for development in a certain province, and its development potential is sufficient.

### 3.3. Current Status of Coal Energy Consumption

According to the information on relevant websites, China's coal industry has made some progress in recent years, and there are still some unpredictable problems in the development process. We need to solve this problem fundamentally. Although the industrial structure and the energy structure are developing steadily, it still cannot change the dominant position of coal in the energy structure. During the “13th Five-Year Plan” period, the pollutants generated by coal consumption will bring a series of problems to the environment, climate, ecology, and resources. Therefore, the total coal consumption must be controlled, laying the foundation for subsequent consumption forecast analysis.

A certain city obtained initial data from the “Investigation Form for Coal Use in Key Energy-Using Units” issued by the Energy Conservation Supervision Center and the 2020 Statistical Yearbook issued by the Municipal Bureau of Statistics. We then compared and verified the data to determine. The final survey data *A*: 18.763 million tons of standard coal; the city's coal consumption statistics of enterprises above designated size in 2020 obtained from the statistical yearbook released by the Bureau of Statistics *B*: 17.256,200 tons of standard coal; according to the coal consumption data structure of the Bureau of Statistics in the past few years, the data gap between *B* and *A* is predetermined as the consumption data of small industrial enterprises and the construction industry under the designated size *C*: 1.5068 million tons of standard coal.

**Figure 5:**
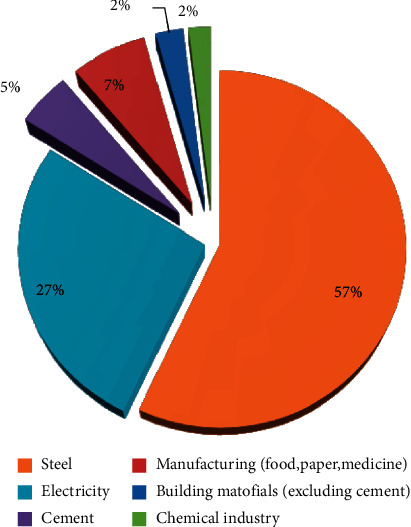
Coal consumption in various industries in a certain city in 2020.

Figure 5 shows the annual coal consumption of various industries in a certain city in 2020.

During the “13^th^ Five-Year Plan” period, we will analyze the growth of energy consumption in China and comprehensively evaluate a city's energy structure adjustment methods, industrial structure adjustment methods, economic development trends, and related policy trends. The setting should continue to grow at a certain rate.  Table 2 is a preliminary estimate of the energy consumption and coal consumption of a certain city's industry.

**Figure 6:**
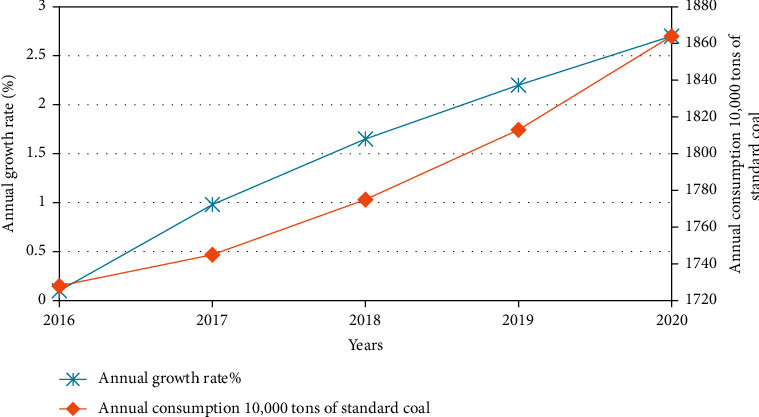
Industrial coal consumption growth trend under the “13th five-year plan” baseline scenario.

It can be seen from Table 2 that under the baseline scenario, the coal consumption of a certain city's industrial sector has increased every year during the “13th Five-Year Plan” period, and the coal consumption in 2016 has increased compared with the coal consumption in 2020.1.3568 million tons of standard coal shows the growth trend, as shown in Figure 6.

It can be seen from Figure 6 that during the “13th Five-Year Plan” period, under the benchmark scenario, the coal consumption rate of a certain city's industrial sector has increased year by year. The annual growth rate of coal consumption in 2020 is 1.66% higher than that in 2016. From this trend, it is necessary to take measures to curb coal consumption.

During the peak period, the energy consumption structure was strictly controlled. Coal was gradually replaced by natural gas and electricity. Crude oil consumption continued to decline. The average annual growth rate of other petroleum products was only 5%. Coal consumption was basically stable from 2015 to 2020, which can be predicted by the model. According to the coal consumption situation, coal consumption reached the highest point in carbon emissions during the “13th Five-Year Plan” period, as shown in Table 3.

### 3.4. Evaluation of Coal Energy Economic Development

The establishment of a special evaluation system for the coal industry in a certain province firstly measures the development level of the coal energy system, economic system, environmental system, and 3*E* system in a certain province's coal industry from 2012 to 2020.  Table 4 shows the calculation results.

**Figure 7:**
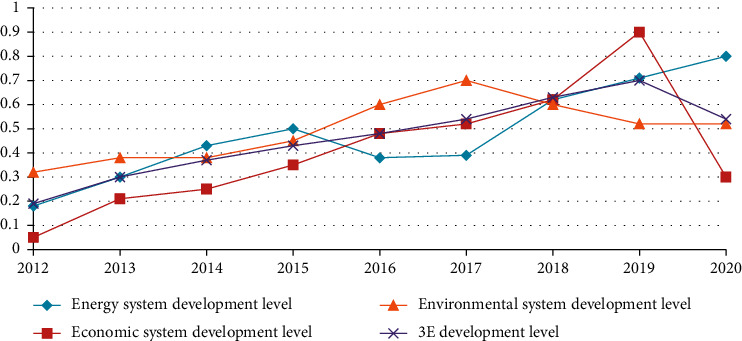
The development trend of the 3*E* system of the coal industry in a certain province.

According to Table 4, the development level of the coal industry system is shown in Figure 7.

It can be seen from Figure 7 that the overall development level of 3E in a province's coal industry system is on the rise. The development level will be the highest in 2020 and the lowest in 2012. The specific analysis is as follows.

It can be seen from Table 4 and Figure 7 that the coal energy subsystem has shown an overall upward trend during the development process from 2012 to 2020. From 2016 to 2017, coal energy has declined, and 2020 is the highest point of development. The energy basis of the coal industry system is the coal energy system, and the development level of the economic system and the environmental system is affected by the development level of the energy system.

## 4. Discussion

### 4.1. Development Countermeasures for New Energy Utilization

In recent years, urban energy consumption is still dominated by coal consumption, the proportion of renewable energy and new energy consumption is low, energy consumption is simplistic, and the energy consumption structure is unreasonable. There is still a big gap with the national energy development plan. To change the current energy consumption structure that relies on coal, it is necessary to focus on the development of alternative energy sources, mainly using clean, environmentally friendly renewable energy sources to replace the traditional energy sources that are polluting, high in emissions, and nonrenewable. To this end, a city must study and formulate strategic goals and policy measures in line with local energy development strategies in accordance with the various energy policies formulated by the state and autonomous regions in combination with its own reality and require a city to develop and utilize renewable energy and new energy on a large scale. Actively do a good job in policy publicity and implementation to lay a solid foundation for achieving the established development goals.

At the national level, China conducts unified planning for the renewable energy and new energy markets, correctly uses market functions to allocate resources, promptly introduces competition mechanisms, accelerates cost reduction and enhances the vitality of the renewable energy and new energy markets. China needs to establish investment and financing mechanisms such as fund support, enterprise raising, and social participation, build a cooperation platform between banks and enterprises, and open up direct financing channels. Each city needs to be guided by the national policy mechanism to speed up the formulation of specific support policies and at the same time provide special financial subsidies to the city for the development and utilization of renewable energy and new energy, adopting various forms such as direct subsidies, subsidies on behalf of rewards, and partial subsidies, to ensure the smooth implementation of renewable energy and new energy development and utilization demonstration projects. At the same time, laws and regulations that encourage and support the development of renewable energy and new energy should be improved as soon as possible, so that renewable energy and new energy development and utilization projects can be followed by law and relevant legislation should be formulated with reference to national laws and regulations and industry technical standards.

The new energy industry has high technical requirements, and a city should speed up the establishment of a development mechanism that combines government leadership and market power to build a renewable energy and a new energy innovation system. A city can rely on its unique advantages in renewable energy and new energy storage, with the help of national and provincial engineering technology centers related to renewable energy and new energy, and combine technology resources in the field of renewable energy and new energy to attract renewable energy and new energy demonstration project. At the same time, the city should support universities in the region to establish disciplines in the field of renewable energy and new energy, cultivate a group of renewable energy and new energy talents, establish a group of renewable energy and new energy engineering technology centers, key laboratories, and other professional institutions and a batch of renewable energy and new energy approved companies. Through internal training and external recommendation of renewable new energy talents, implement human resource strategies, establish a regional renewable new energy expert database, and improve overall innovation capabilities and levels. The city should also actively carry out renewable energy and new energy technology exchange activities, strengthen technology docking, accelerate the transformation of results, promote the industrialization of science and technology, and ultimately accelerate technological reform and industrial innovation, guide the development and utilization of renewable energy and new energy, and control renewable energy and new energy product market access standards, continuous improvement of the industrial chain, establishment of a good competitive market, and elimination of outdated production capacity.

### 4.2. Coal Energy Consumption Control Measures

Under the coal management scenario, electricity consumption in a certain city will gradually increase in 2020. Due to the unique power structure of a certain city, it is necessary to reduce the proportion of coal electricity consumption, optimize the electricity consumption structure of a certain city, and introduce new electricity consumption. The power generation technology of the United States reduces the amount of coal used from carbon sources, and most of them use clean energy such as wind power, solar energy, and nuclear power to replace coal for power generation. In the short term, due to changes in the power structure, a city will be heavily dependent on outsourced power. This process may last for 10 to 20 years, but in the long run, changes in the power structure will definitely help the economy of some cities. Development and improvement of the environment: According to the overall situation of the city's industry, combined with the current situation at home and abroad, the coal-fired power generation companies in a city were investigated, analyzed the possibility of these companies to reduce coal consumption, and proposed corresponding coal reduction measures. According to the survey of coal consumption in the city's power industry, the total coal consumption of the three major enterprises, Huayuan Power Plant, Changyuan Power Plant, and Qingshan Thermal Power Plant, basically accounted for more than 95% of the total coal consumption of a certain city's power industry. Other thermal power plants are mainly garbage power plants. Due to the small scale of the company, the possibility of saving energy and reducing coal is unlikely, so we will not consider it for the time being.

Reducing the integrated line loss is to minimize the power loss caused by wiring and other reasons. Therefore, we must try to avoid energy loss. One is to improve the wire itself and choose the cross section and material of the wire reasonably according to the economic current density method to ensure the quality of the wire. People should make reasonable wiring, shorten the length of wires, regularly check and replace aging wires; install a static reactive power compensator according to some principles of static reactive power compensation; reasonably select the capacity of the asynchronous motor to match the economic benefits and load to reduce unnecessary power.

Actively promote energy-saving equipment and improve the efficiency of terminal power consumption. Through research on typical energy-saving technologies such as energy-saving lamps, high-efficiency motors, and energy-saving transformers in China, it is found that high-efficiency energy-saving equipment has huge development potential. Home appliance products cover a wide area and are used in a large amount. Through the implementation of economic subsidy policies, users can trade in and use old energy-saving air conditioners, microwave ovens, ovens, and other electrical equipment, which can improve the efficiency of terminal power consumption.

### 4.3. Countermeasures for the Development of the Coal Energy Economy

One-third of the land in a province contains coal energy, accounting for one-fourth of China's coal energy supply. There are two different types of coal: coking coal and anthracite. The province has unique mining conditions and geographic advantages. In the process of developing the coal industry, it is necessary to build a complete ecological industry value chain based on the principles of material circulation, energy flow and information transmission in the ecosystem from the three levels of product production companies, symbiotic enterprises, and industrial parks. A certain province should attach importance to the sustainable use of coal resources and the coordinated development of the environment and formulate a sustainable development strategy from the social, economic, technological, and environmental aspects. First, make full use of coal resources, then gradually eliminate extensive production operations, and finally actively exploit solar energy, nuclear power, hydropower, wind power, and other clean energy sources. The promulgation of coal energy policies can implement macro-control from the political and legal levels and correctly guide the development and utilization of coal energy. The coal industry in a certain province resolutely implements the “Opinions of the People's Government of a Province on Promoting the Integration and Paid Utilization of Resources in Coal Enterprises” and the “Implementation Opinions on the Integration and Transformation of Resources in the Coal Industry in a Province,” guided by scientific development and China's coal mine ecological environment management. The plan is to guide, formulate, and implement a “concentrated pollution control system,” and continuously improve the defects in the relevant policies of the coal industry.

Due to some market reasons, transportation, and related enterprise disputes, coal companies often face the scene of excessive product, resulting in a large accumulation of coal resources. Due to the strong dependence on external market demand, the demand often causes the coal price in a certain province to drop. There is an oversupply situation. The province should continue to promote investment promotion, actively support the construction of enterprises to broaden the coal industry chain, explore new ways for coal energy utilization, solve the contradiction between technology and economy, be innovation-oriented, take environmental protection as the goal, adopt a circular economy model and promote 3*E* complexity. The exchange of personnel, technology, and other elements between the system and the external environment truly realizes the rational use of coal resources. At the same time, through the study of relevant literature, it is known that the advancement of science and technology and the application of control methods may break the limit of ecological carrying capacity and promote the qualitative leap of the 3*E* complex system.

## 5. Conclusion

The method in this article is mainly based on the overall new energy utilization decision-making of a certain city. Due to the differences in the types, reserves, and development and utilization of new energy in different areas of the city, the decision-making process for the development and utilization of renewable energy and new energy in each region still needs to be combined with the local areas. The study is to formulate targeted policies and measures in line with the sustainable development of energy, economy, and environment in the region. Then it investigated the development of coal energy economy in a certain province. The theory of circular economy and sustainable development has important guiding significance for the coordinated development of the coal industry system. In recent years, the theory of circular economy and the theory of sustainable development have become the focus of attention in the field of coal industry research. Since a large amount of accompanying organisms and wastes will be released from the mining and utilization of coal energy to the conversion of consumption, the only way to go is sustainable development. We should comprehensively address the coordinated development of the coal energy system, the economic system, the environmental system, and the coal industry 3E system.

## Figures and Tables

**Table 1:** The total contribution rate of various new energy sources to GDP from 2001 to 2020.

Years	Hydropower	Wind energy	Biomass energy
2001	2.5971818	6.47766410	0.288565754
2002	8.4105063	−1.08957042	0.101496280
2003	10.1432896	41.79817055	0.722989087
2004	−3.8896444	−13.70992193	0.145704893
2005	−1.9010146	−1.62535695	0.08388528
2006	−0.2491803	−0.84301982	0.055147777
2007	2.6912122	−1.44095706	0.143262026
2008	0.3754298	−0.45896078	0.09815149
2009	0.3596158	−0.24135777	0.011113961
2010	−0.6797325	−0.14687575	0.047450224
2011	−2.2055192	−0.43278606	0.027715456
2012	0.9550422	−0.23611210	0.015367445
2013	1.903172	−0.67924593	0.051597658
2014	9.9471554	−1.23977397	0.156730455
2015	2.3206587	−0.06921151	0.016057095
2016	−2.9267123	−0.09815641	0.014487874
2017	4.2160685	0.10347749	0.040035213
2018	−3.8833657	−0.18907169	0.005590534
2019	0.7041158	0.19185754	0.069807545
2020	0.1167582	−1.25717218	1.567259814

**Table 2:** Coal consumption of industrial coal above designated size during the 13th five-year plan period in a certain city (10,000 tons of standard coal).

Years	Total energy consumption	Proportion of coal (%)	Coal consumption
2016	4335.83	39.83	1727.398
2017	4552.64	38.30	1744.112
2018	4780.25	37.08	1772.997
2019	5019.26	36.11	1812.95
2020	5270.23	35.34	1863.08

**Table 3:** Coal consumption under the carbon emission peak scenario.

Years	2015	2016	2017	2018	2019	2020
Coal consumption/10,000 tons of standard coal	1725.61	1770.54	1791.70	1791.07	1770.58	1732.06

**Table 4:** Evaluation results of the coordinated development level of a province's coal industry from 2012 to 2020.

Years	Coal energy system development level (Ee)	Economic system development level	environmental system development level	3*E* development level (*E*^*∗*^)
2012	0.1668	0.0460	0.3184	0.1771
2013	0.2928	0.2066	0.3634	0.2876
2014	0.4246	0.2552	0.3701	0.3500
2015	0.4811	0.3561	0.4652	0.4340
2016	0.3801	0.4844	0.5847	0.4832
2017	0.3903	0.5267	0.7026	0.5401
2018	0.6451	0.6405	0.5935	0.6264
2019	0.7198	0.8877	0.5163	0.7078
2020	0.7971	0.2880	0.5193	0.5347

## Data Availability

The data used to support the ﬁndings are available from the corresponding author upon request.

## References

[1] InterAcademy Council (2007). *Lighting the Way: Toward a Sustainable Energy Future*.

[2] Git P. (1980). *A Symposium on World Energy Prospects*.

[3] Brandt J., Silver J. D., Christensen J. H. (2011). Assessment of health-cost externalities of air pollution at the national level using the EVA model system. *CEEH Scientific Report No 3, Centre for Energy, Environment and Health Report Series*.

[4] US Environmental Protection Agency (2018). *Environmental Benefits Mapping and Analysis Program (BenMAP)*.

[5] Flachs E. M., Bønløkke J. H., Sigsgaard T., Chen Y. (2012). *Description of theHIAline in the CEEHintegratedmodelling Chain,” CEEHScientific Report No. 5*.

[6] Brandt J., Silver J. D., Frohn L. M. (2012). An integrated model study for Europe and North America using the Danish Eulerian hemispheric model with focus on intercontinental transport of air pollution. *Atmospheric Environment*.

[7] Juel K., Sørensen J., Brønnum-Hansen H. (2008). Supplement: r. *Scandinavian Journal of Public Health*.

[8] Al Shidi H., Sulaiman H., Amoatey P. (2016). Shifting to renewable energy to mitigate carbon emissions: initiatives by the states of gulf cooperation council. *Low Carbon Economy*.

[9] Zhou H. C., Song Z. H., Liu Y. F., Zhang X. L. (2019). Evaluation, comparison and improvement of ecological civilization construction evaluation index system. *Eco-Economy*.

[10] Zhilin L., Gong X., Chen J. (2020). Functional requirements of systems for visualization of sustainable development goal (SDG) indicators. *Journal of Geovisualization and Spatial Analysis*.

[11] Li L., Dong S. S., Wen X. M. (2006). An energy efficient clustering routing algorithm for wireless sensor networks. *The Journal of China Universities of Posts and Telecommunications*.

[12] Hou Y. T., Shi Y., Pan J., Midkiff S. F. Lifetime-optimal data routing in wireless sensor networks without flow splitting.

[13] Gupta G., Younis M. Load-balanced clustering of wireless sensor networks.

[14] Steere D. C., Baptista A., McNamee D., Pu C., Walpole J. Research challenges in environmental observation and forecasting systems.

[15] Liu Y.-Y., Ji H., Yue G.-X. (2006). Routing protocol with optimal location of aggregation point in wireless sensor networks. *The Journal of China Universities of Posts and Telecommunications*.

